# Periphytic Algae and Cyanobacteria from the Rio Doce Basin Respond Differently to Metals and Salinity, Showing Different Potential for Bioremediation

**DOI:** 10.3390/plants10112349

**Published:** 2021-10-30

**Authors:** Marcelo Pedrosa Gomes, Letícia Yoshie Kochi, Patrícia Lawane Freitas, Cleber Cunha Figueredo, Philippe Juneau

**Affiliations:** 1 Laboratório de Fisiologia de Plantas sob Estresse, Departamento de Botânica, Setor de Ciências Biológicas, Universidade Federal do Paraná, Avenida Coronel Francisco H. dos Santos, 100, Centro Politécnico Jardim das Américas, C.P. 19031, Curitiba 81531-980, Brazil; leticiayoshie96@gmail.com (L.Y.K.); patricialawane@gmail.com (P.L.F.); 2 Departamento de Botânica, Instituto de Ciências Biológicas, Universidade Federal de Minas Gerias, Avenida Antônio Carlos, 6627, Pampulha, C.P. 486, Belo Horizonte 31270-901, Brazil; clebercf@icb.ufmg.br; 3 Ecotoxicology of Aquatic Microorganisms Laboratory, GRIL, EcotoQ, TOXEN, Department of Biological Sciences, Université du Québec à Montréal, Montréal, QC H3C 3P8, Canada

**Keywords:** iron, manganese, sodium chloride, periphyton, stress

## Abstract

We have studied the isolated and combined effects of metals (Fe and Mn) and NaCl the on growth, physiology, and metal-uptake capacity of two photosynthetic periphytic species—*Synechococcus elongatus* (Cyanobacteria) and *Chlorococcum infusionum* (Chlorophyta)—isolated from an impacted area of the Rio Doce River (Brazil) after the Fundão dam collapse. The effective concentrations found to reduce 10 and 50% growth were 15.2 and 31.6 mg Fe L^−1^, and 2.5 and 7.9 mg Mn L^−1^ for *S. elongatus* and 53.9 and 61.6 mg Fe L^−1^, and 53.2 and 60.9 mg Mn L^−1^ for *C. infusionum*. Although the metal toxicity was related to oxidative stress, both species showed activation of antioxidant systems under phytotoxic concentrations of Fe and Mn. By binding large concentrations of metals on its cell surface and thus avoiding their entrance into the cells, *C. infusionum* presents greater resistance to Fe and Mn than *S. elongatus*. Under environmental realistic concentrations of Fe and Mn in river water from the Rio Doce Basin, *S. elongatus* and *C. infusionum* showed a metal removal efficiency of 42 and 65% and 53 and 79%, respectively after 96 h. These species were insensitive to increased NaCl concentrations which, in addition, did not disrupt the metal removal capacity of the species. Due to their salt and metal tolerance, *S. elongatus* and *C. infusionum* can be used for the remediation of waters contaminated with Fe and Mn.

## 1. Introduction

On 5 November 2015, the Fundão dam operated by the company Samarco collapsed in the municipality of Mariana, Minas Gerais (Brazil), releasing about 50 million m^3^ of tailings in the Rio Doce Basin. The tailings impacted more than 600 km downstream to the Rio Doce Basin [1], reaching the estuary, causing an immediate impact on the local native vegetation of the Atlantic Forest and aquatic ecosystems, and significantly increasing the background trace metal contents of sediments [2].

Several environmental reports point to the presence of a significant amount of trace elements in the Rio Doce water after the disaster [3,4], with lead (Pb), arsenic (As), copper (Cu), manganese (Mn), and iron (Fe) concentrations exceeding the maximum permissive value by Brazilian legislation (being 0.033, 0.033, 0.2, 0.5, and 1.4 mg L^−1^, respectively). However, the sediment and water assessment carried out by the Geological Service of Brazil, CPRM [5], did not relate the water contamination with the disaster, since the concentrations of trace-elements in the water after the disaster were similar to those previously recorded. In any case, the mining activity near the river and streams that form the Rio Doce River is responsible for the continuous introduction of trace elements in waters, which may affect environmental quality.

Freshwater salinization is a global concern and has been attributed to agriculture, resource extraction, and land clearing [6]. Moreover, sodium-based organic reagents, such as sodium diethyldithiocarbamate (DDTC), are widely used in the process of metal ore flotation [7]. High concentrations of sodium (Na) were detected in sediments of areas affected by the mud from the Fundão dam, and Na was proposed as one of the main candidates responsible for the mud’s environmental toxicity [8]. In this context, due to its mining-activity influence as well as the disposal of iron tailing in its water, Rio Doce is prone to salinization; consequently, the combined contamination of metals and NaCl must be expected. This is also a worldwide problem since several bodies of water around the world are threatened by similar conditions [9], which may alter their ecosystem services. The mechanisms of combined contamination by metals and increased salinity in freshwater cyanobacterial and algal species, however, are poorly understood.

Periphytic communities serve as a vital component of stream ecosystems, playing an intermediate role between the overlaying water column and the substrate beneath [10]. In addition to acting as a link in the transfer of energy along the food chains, periphytic species also act as bioindicators of water quality and ecological health [10]. Periphytic cyanobacteria and green algae often exhibit considerable metal and salinity tolerance [11,12,13,14,15]. However, the metabolic costs of metal or salt tolerance may reduce growth, and indeed, the growth rates of several species decrease as metal and salt concentrations increase, but in a species-dependent way [11,12,13,14,16]. Metal stress is related to the metal-induced oxidative stress, through reactive oxygen species (ROS) generation [12], which can disrupt photosynthesis and energetic metabolism. Decreased photosynthesis is also observed in freshwater species exposed to increased salt concentrations [13,17]. By studying two green algal species, Singh and collaborators [18] observed that high salt levels resulted in inhibition of cell division, probably as a result of lower chlorophyll (Chl) content due to ROS induced chlorosis, photoreduction, and formation of triplet Chl, with damages to PSI and PSII. Therefore, metals and NaCl can have synergic or additive effects on cyanobacterial and algal physiology, although little is known about the interaction of these two factors. 

The ability of microalgal community to grow in polluted sites also results in the improvement of water quality [19]. Indeed, due to their capacity to remove contaminants such as metals and inorganic and organic toxic substances from water (including wastewaters), cyanobacteria and algae have been efficiently employed for bioremediation purposes [19]. However, very few studies have been conducted to understand the effect of increasing salinization in the natural ability of periphytic cyanobacteria and algae to reclaim metals from water. Since NaCl affect their physiology, it may also play a role in their bioremediation capacity. Therefore, here, we studied the isolated and combined effects of metals (Fe and Mn) and NaCl on growth, physiology, and metal-uptake capacity of two periphytic photosynthetic species—*Synechococcus elongatus* (Cyanobacteria) and *Chlorococcum infusionum* (Chlorophyta)—isolated from an impacted area of the Rio Doce River after the Fundão dam collapse. In addition to their responses to Fe and Mn, we aimed to elucidate if there is a relationship between metal tolerance and the strategies for metal accumulation and to determine if the water salinization can constrain the ecological benefits displayed by the periphytic species. 

## 2. Results

### 2.1. Mn and Fe Effects and Uptake in S. elongatus 

**Figure 1:**
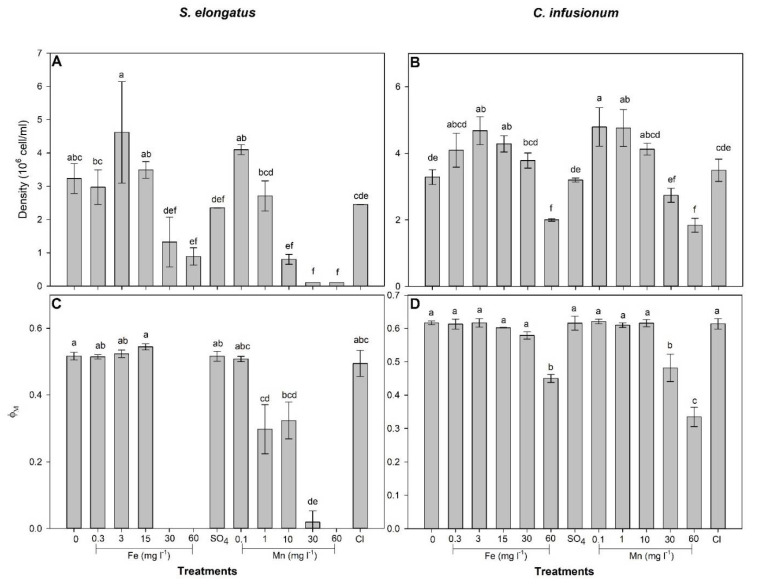
Cell density (**A**,**B**) and maximal PSII photochemical yield (Φ_M_) (**C**,**D**) in *Synechococcus elongatus* and *Chlorococcum infusionum* exposed to increased concentrations of Fe or Mn (mg L^−1^) or to 103.20 mg SO_4_ L^−1^ and 77.45 mg Cl L^−1^ for 96 h. Bars represent means ± SD of six replicates. Different letters indicate significant difference (*p* > 0.05) by the post hoc Tukey test.

After 96 h of exposure, the *S. elongatus* cell density was increased by 3 mg Fe L^−1^ treatment and decreased by Fe concentrations > 30 mg L^−1^ and Mn concentrations ≥ 10 mg L^−1^ in relation to the control (F = 22.79; *p* < 0.01; Figure 1). No significant effects of SO_4_ and Cl were observed on cell density in relation to the control (*p* > 0.05). The calculated EC_10_ and EC_50_ for Fe and Mn were 15.2 and 31.6 mg Fe L^−1^, and 2.5 and 7.9 mg Mn L^−1^, respectively. For concentrations up to 15 mg L^−1^, compared to the control, Fe had no significant impact on the maximal PSII photochemical yield (Φ_M_) after 96 h exposure but completely inhibited Φ_M_ at concentrations ≥30 mg L^−1^ (F = 33.87; *p* < 0.0001; Figure 1). Φ_M_ was gradually decreased by Mn for concentrations ≥1 mg L^−1^ and was completely inhibited at a concentration of 60 mg Mn L^−1^ (Figure 1). 

**Figure 2:**
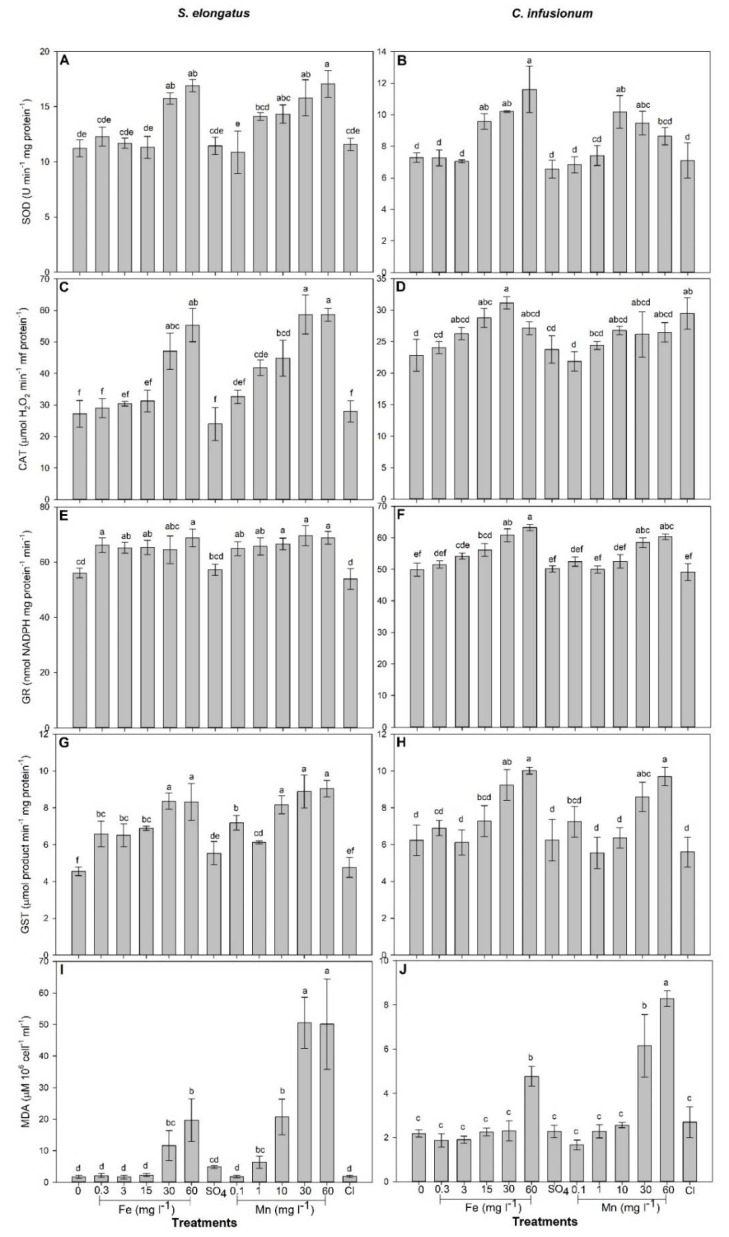
The activities of superoxide dismutase (SOD) (**A**,**B**), catalase (CAT) (**C**,**D**), glutathione reductase (GR) (**E**,**F**), glutathione-*S*-transferase (GST) (**G**,**H**), and the concentration of malondialdehyde (MDA) (**I**,**J**) in *Synechococcus elongatus* and *Chlorococcum infusionum* cells exposed to increased concentrations of Fe or Mn (mg L^−1^) or to 103.20 mg SO_4_ L^−1^ and 77.45 mg Cl L^−1^ for 96 h. Bars represent means ± SD of three replicates. Different letters indicate significant difference (*p* > 0.05) by the post hoc Tukey test.

In relation to control, SOD (F = 16.59) and CAT (F = 27.61) activity as well as MDA concentration (F = 32.21) increased for cells exposed to Fe and Mn concentrations ≥30 mg L^−1^ and ≥1 mg L^−1^, respectively, in relation to control (*p* < 0.0001; Figure 2). Regardless of the concentration, GR (F = 8.78) and GST (F = 20.57) activity was increased by Fe and Mn treatments (*p* < 0.001; Figure 2).

The total removal of Fe (F = 94.50) increased as the metal concentration increased in the growth solution (Table 1). The external (C_extra_) and internal (C_intra_) concentrations of Fe increased in cells treated with Fe concentrations ≥3 mg L^−1^ in relation to control, being the greatest in cells treated with 30 and 60 mg Fe L^−1^ (F = 52.10 and 14.25, respectively; Table 1). No Mn was detected in control cells (Table 2). Total removal (F = 12.26) as well as the C_extra_ (F = 12.71) and the C_intra_ (F = 13.65) of Mn were greater in cells treated with Mn concentrations ≥10 mg L^−1^ in relation to those treated with 0.1 mg Mn L^−1^ (F = 12.26; Table 2). Among cells treated with Fe, the removal efficiency (RE) was greater in those exposed to 30 mg Fe L^−1^ and did not significantly differ for other Fe treatments (F = 14.25; Table 1). RE was decreased as Mn concentration in the solution increased (F = 64.68; Table 2). 

### 2.2. Mn and Fe Effects and Uptake in C. infusionum

After 96 h of exposure, *C. infusionum* cell density was increased when treated with 3 and 15 mg Fe L^−1^ as well as with 0.1 and 1 mg Mn L^−1^, while a decrease was observed when this species was exposed to 60 mg L^−1^ of Fe and Mn (*p* = 25.62; *p* < 0.001; Figure 1). The calculated EC_10_ and EC_50_ for Fe and Mn were 53.9 and 61.6 mg Fe L^−1^, and 53.2 and 60.9 mg Mn L^−1^, respectively. Φ_M_ was decreased in algal cells exposed to 60 mg Fe L^−1^ and Mn concentrations ≥30 mg L^−1^ (F = 75.36; *p* < 0.0001; Figure 1). 

In relation to control, SOD activity increased in cells exposed to Fe concentrations ≥15 mg L^−1^ and in cells exposed to 10 and 30 mg Mn L^−1^ (F = 15.21; *p* < 0.0001; Figure 2). On the other hand, increased CAT activity was only observed in cells exposed to 15 and 30 mg Fe L^−1^ (F = 6.66; *p* < 0.0001; Figure 2). GR (F = 26.05) and GST (F = 13.02) activities significantly increased (*p* < 0.0001) in cells exposed to Fe concentrations ≥15 mg L^−1^ and ≥30 mg L^−1^, respectively, and to Mn concentrations ≥30 mg L^−1^ in relation to control (Figure 2). MDA concentration was greater in cells exposed to 60 mg Fe L^−1^ and to Mn concentrations ≥ 30 mg L^−1^ in relation to the control (Figure 2).

The total removal (F_Fe_ = 94.50; F_Mn_ = 388.83) as well as C_extra_ (F_Fe_ = 253.98; F_Mn_ = 257.43) and C_intra_ (F_Fe_ = 162.56; F_Mn_ = 113.74) of Fe and Mn increased as the metal concentration in the growth solution increased (Table 1 and Table 2). Among cells treated with Fe, RE was greater in those exposed to 30 mg Fe L^−1^ and did not significantly differ from other Fe-treatments (F = 9.95; Table 1). Mn-RE was the greatest in cells exposed to 1 mg Mn L^−1^ and the lowest in cells exposed to 60 mg Mn L^−1^ (F = 39.12; Table 2). No Mn was detected in cells of control treatments (Table 2).

### 2.3. NaCl and Metal (Fe and Mn) Combined Effects on S. elongatus and C. infusionum

**Figure 3:**
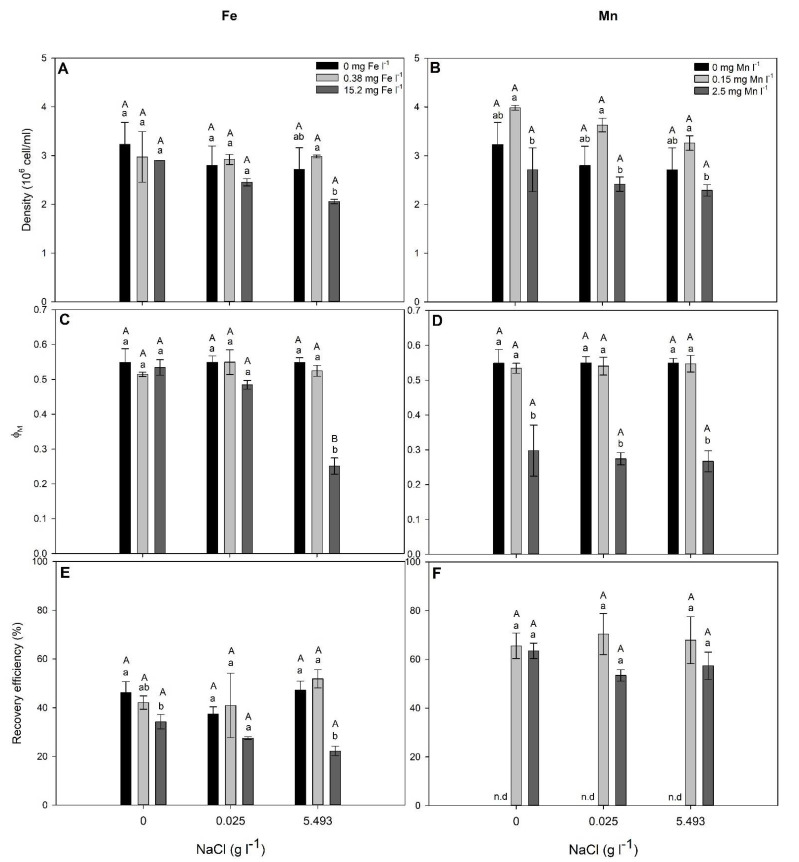
Cell density (**A**,**B**), maximal PSII photochemical yield (Φ_M_) (**C**,**D**) and Fe- and Mn-recovery efficiency (**E**,**F**) in *Synechococcus elongatus* exposed to different concentrations of NaCl (g L^−1^) with addition of 0, 0.38 and 15.2 mg Fe L^−1^ or 0, 0.15 and 2.5 mg Mn L^−1^. Bars represent means ± SD of three replicates. Lowercase letters indicate significant differences among Fe or Mn concentrations within the same NaCl addition, while uppercase letters indicate significant differences between NaCl concentrations within the same Fe or Mn addition, by the post hoc Tukey test (considering *p* < 0.05). n.d = not determined.

NaCl alone did not significantly affect (*p* > 0.05) the cell density, Φ_M_ and Fe-RE of *S. elongatus* (Figure 3, Table 3). When treated with 5.493 g NaCl L^−1^, the density of Φ_M_ and Fe-RE were decreased in cells exposed to 15.2 mg Fe L^−1^ in relation to those exposed to 0 and 0.38 mg Fe L^−1^ (Figure 3). Among cells exposed to Mn, greater density was observed in those treated with 0.15 mg Mn L^−1^, and a lower Φ_M_ was observed in those treated with 2.5 mg Mn L^−1^, regardless of the NaCl concentration in the growth media (Figure 3). Mn-RE was not significantly affected (*p* > 0.05) by both Mn and NaCl concentration (Figure 3; Table 3).

**Figure 4:**
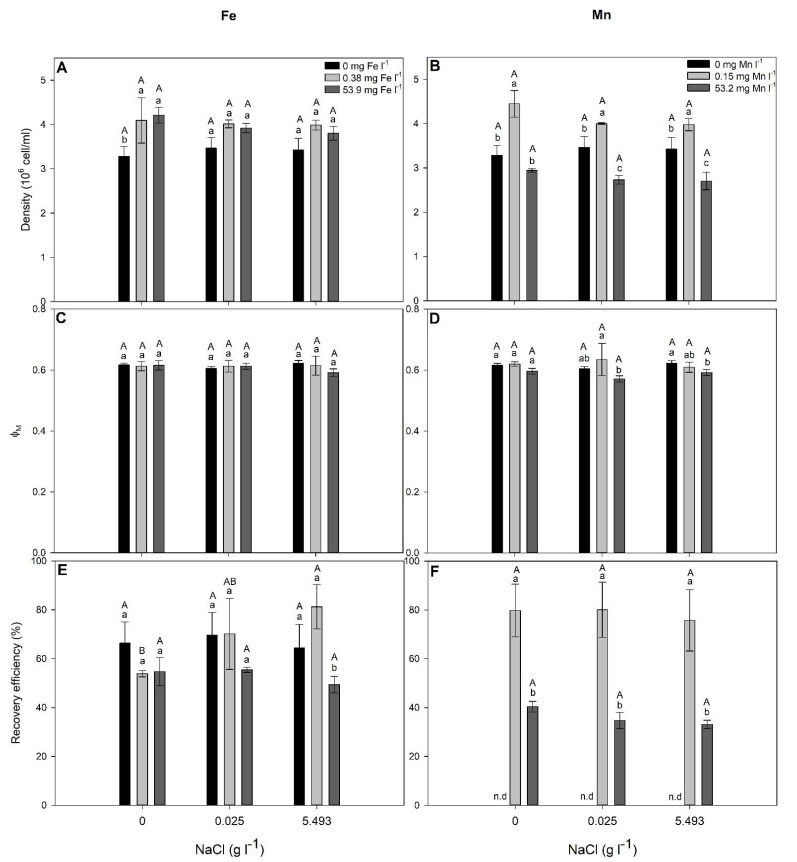
Cell density (**A**,**B**), the maximal PSII photochemical yield (Φ_M_) (**C**,**D**) and the Fe- and Mn-recovery efficiency (**E**,**F**) in Chlorococcum infusionum *exposed to increased concentrations of NaCl (g L^−1^) with addition of 0, 0.38, and 15.2 mg Fe L^−1^ or 0, 0.15, and 2.5 mg Mn L^−1^*. Bars represent means ± SD of three replicates. Lowercase letters indicate significant differences among Fe or Mn concentrations within the same NaCl addition, while uppercase letters indicate significant differences between NaCl concentrations within the same Fe or Mn addition, by the post hoc Tukey test (considering *p* < 0.05).

As for *S. elongatus*, NaCl alone did not significantly affect (*p* > 0.05) the cell density and Fe-RE of *C. infusionum* (Figure 4, Table 3). In the absence of NaCl, the cell density was increased by Fe presence and by 0.15 mg Mn L^−1^ (Figure 4). When exposed to NaCl, the cell density and Φ_M_ were not changed by Fe-exposure but decreased for the highest Mn concentration in relation to cells not treater with Mn (Figure 4). When treated with 5.493 g NaCl L^−1^, the Fe-RE decreased in cells exposed to 53.9 mg Fe L^−1^ in relation to those exposed to 0 and 0.3 mg Fe L^−1^ (Figure 3). Moreover, in cells treated with 0.38 mg Fe L^−1^, the Fe-RE was increased by the treatment with 5.493 g NaCl L^−1^ in relation to 0 g NaCl L^−1^ (Figure 4). The Mn-RE was not significantly affected by NaCl but decreased in cells exposed to 53.2 mg Mn L^−1^ (Figure 4).

## 3. Discussion

Iron and manganese fall into the group of trace elements that function as micronutrients for photosynthetic microorganisms, being involved in numerous physiological processes. Therefore, as observed here, at low concentrations, Fe and Mn can stimulate growth by increasing cell density (Figure 1). However, at high concentrations, these metals become toxic, impairing metabolism and growth [20], although information about micronutrient effects on photosynthetic microorganisms in the literature is more related to nutrient limitation conditions than to an excess of metals in water [21,22,23]. Indeed, with the exception of contaminated or naturally enriched systems (i.e., volcanic areas), an excess of metals in the aquatic ecosystems is not observed. However, anthropic activities have been responsible for increasing metal concentrations in aquatic systems. Beyond rock weathering, three anthropic sources (mining and manufacturing, fertilizer and pesticide use, and waste discharge) have been the major causes of metal pollution in rivers and lakes of South America during the last decades [24], and disasters such as the one in Mariana may have increased, at least in the short term, the metal concentrations in water. Therefore, investigations on the responses of aquatic organisms to excessive metal concentrations are claimed. 

Photosynthetic microorganisms are particularly responsive to metal in water [25,26,27]. Since our study was conducted with species isolated from an area with a history of water metal contamination, it is not a surprise to see that deleterious effects of Fe (EC_10_ = 15.2 and 53.9 for *S. elongatus* and *C. infusionum*, respectively) and Mn (EC_10_ = 2.5 and 53.2 for *S. elongatus* and *C. infusionum*, respectively) were only observed at concentrations much higher than that observed in their occurrence area (up to 7.3 mg L^−1^ of total Fe and 0.57 mg Mn L^−1^) [28]. It seems that microalgae species or ecotypes living in metal contaminated areas have been adapted to metal toxicity and evolved metal tolerance [29,30]. Moreover, several algal species are known for their potential to grow under extreme metal contamination conditions which are related to their efficient defense strategies against the toxic effects of metals [31]. The greater EC_10_ and EC_50_ observed for *C. infusionum* indicate that this green alga is more tolerant to Fe and Mn than the cyanobacteria *S. elongatus*. Different groups of photosynthetic microorganisms are known to exhibit varying levels of tolerance to metals. Indeed, similarly to our results, greater tolerance to Cu, Cd, Cr, and Zn was observed among members of the Chlorophyceae than of Cyanobacteria [32,33,34]. Biochemical and physiological mechanisms are particularly involved in tolerance in the case of an excess of micronutrients [30]. Due to their redox potential, excessive intracellular concentrations of metals induce oxidative stress, with deleterious effects on primary metabolism which results in decreased growth [31]. Our results indicate, for both studied species exposed to metals, that increased oxidative stress (as evaluated by MDA concentration, an indicator of lipid peroxidation) was followed by decreased photosynthesis and growth (Figure 1 and Figure 2). In both species, increased activity of antioxidant systems (SOD, CAT, GR, and GST) was observed in cells exposed to metal concentrations leading to reduced photosynthesis and growth (Figure 1 and Figure 2). Therefore, differences of metal tolerance among the studied species cannot be explained by the lack of antioxidants to avoid metal toxicity. So, how can the great metal tolerance of *C. infusionum* in relation to *S. elongatus* be explained?

Biosorption is one of the foremost methods employed by algae for metal resistance [31]. Through this process, microalgae prevent the uptake of metal ions into the cell interior [31], avoiding the deleterious effects on cell metabolism of excessive metal concentration. The microorganism cell surface acts as an efficient sink for metals due to the presence of large numbers of anionic groups which favor interactions with the cationic metal ions [35]. Our results indicate that *C. infusionum* allocate much more Fe and Mn to the cell surface than *S. elongatus* (Table 1 and Table 2). The C_extra_ of Fe for *S. elongatus* ranged from 29 to 38% of the total removed metal, being 38 and 37% in cells treated with 30 and 60 mg Fe L^−1^, respectively, where deleterious effects to photosynthesis and growth were more accentuated (Figure 1). For *C. infusionum* C_extra_ of Fe ranged from 33 to 64%, being 62 and 64% of the total removed metal in cells treated with 30 and 60 mg Fe L^−1^. Similarly, while the C_extra_ of Mn in *S. elongatus* ranged from 26 to 38% of the total removed metal in toxic concentrations of the metal (≥10 mg Mn L^−1^), it varies from 57 to 65% in *C. infusionum*. Variation in the cellular composition (mainly cell wall) among microbial systems is the major cause of different abilities and specificity for metal binding [36]. Therefore, by binding large concentrations of metals on its cell surface and avoiding their entrance into the cells, *C. infusionum* presents greater resistance to Fe and Mn than *S. elongatus*.

Although *C. infusionum* showed similar EC_10_ for Fe and Mn, *S. elongatus* was much more sensitive to Mn (EC_10_ = 2.5 mg L^−1^) than Fe (EC_10_ = 15.2 mg L^−1^). Both metals present two similar oxidation states and chemical behaviors, being involved in oxidative stress (as observed by increased lipid peroxidation, Figure 2). The weaker stress induced by Fe in relation to Mn, therefore, can be associated with the greater cell necessity of Fe than Mn. Iron is much more required for metabolisms and growth in addition to being preferably taken up by microalgae than Mn [37]. In addition, given their evolutionary origin in an Fe-rich anoxic ocean, cyanobacteria often show great Fe-tolerance [25]. This could justify the greater C_intra_ and removal efficiency of Fe than Mn in cells exposed to 30 and 60 mg L^−1^ (Table 1 and Table 2). In this context, *C. infusionum* is indicated more for Mn removal from water with very high contamination of the metals. Under realistic environmental conditions, both species showed a great ability for Fe and Mn removal from water. For instance, under the highest observed concentration of Fe (0.38 mg L^−1^) and Mn (0.15 mg L^−1^) in water from rivers of the Rio Doce Basin, *S. elongatus* and *C. infusionum* showed a RE of 42 and 65% and 53 and 79%, respectively (Figure 3 and Figure 4), which indicate the ability of both species to be applied for bioremediation purposes.

Secondary salinization (due to anthropogenic activities) is a global and growing threat to aquatic biodiversity [38]. The photosynthetic microorganisms studied here, however, were insensitive to increased concentrations of salt in water (Figure 3 and Figure 4). Cell density and photosynthesis did not statistically change, even when cells were exposed to the highest NaCl concentration found in water of the Rio Doce Basin, demonstrating the salt-tolerance of *S. elongatus* and *C. infusionum*. *Synechococcus* *elongatus* and species of the genera *Chlorococcum* have been previously characterized as halotolerant and able to proliferate in environments with high salt concentrations [39,40]. For instance, the growth of *Chlorococcum* sp. was only significantly inhibited in NaCl concentrations higher than 10 g L^−1^ [39]. Similarly, *S. elongatus* cells tolerate NaCl at concentrations up to 23.37 g L^−1^ (0.4 M) [40,41]. Moreover, as seen here, the Fe and Mn remediation abilities of the studied species were not affected by salinization (Figure 3 and Figure 4). Therefore, even under a perspective of salinization of rivers where these periphytic alga and cyanobacteria occur, their bioremediation capacity to remove Fe and Mn from water is not disturbed by increased NaCl concentrations, which is an important finding when aiming to select microorganisms for bioremediation programs.

## 4. Materials and Methods

### 4.1. Sampling and Collection 

To obtain the cultures, epilithic periphyton was sampled from rocks obtained in the Doce River, at a site that was contaminated by the mining wastes of Fundão (20°11′40″ S/42°51′0.4′ W). This site was chosen because we wanted to study species that were well adapted to the new environmental conditions. The biofilm layer on the rocks was carefully removed by washing the surface with CHU10 medium and by using a soft toothbrush. These liquid samples were collected, transferred to sterile flasks, and maintained at 10 °C until the isolation (the following day). To isolate a single cell for each clonal culture, we performed the traditional micropipette isolation method [42]. The stock cultures of *Synechococcus elongatus (Cyanobacteria)* and *Chlorococcum infusionum (Chlorophyta)* were maintained in a growth chamber illuminated with fluorescent tubes providing a light intensity of 45 ( ± 2.0) µmol photons m^−2^ s^−1^ at 20 (±2.0) °C. The species were cultured in Erlenmeyer flasks under a 12:12 h light:dark regime. The pH was frequently measured and a value of 7.4 was maintained for the appropriate growth of the test microorganisms. All the Erlenmeyers (250 mL) were washed with 10% HCl and rinsed thoroughly with distilled water, prior to use to prevent the binding of metals to the flasks. All the experiments were performed with cultures in their exponential growth phase.

### 4.2. Mn and Fe Effects 

To examine the effect of Fe and Mn on the studied species, sterile Erlenmeyer flasks containing fresh CHU10 medium were inoculated with a respective volume of the stock culture corresponding to 1 × 10^6^ cells. Iron (as FeSO_4_.7H_2_O) and manganese (as MnCl_2_) were added separately to flasks in calculated amount to obtain the final added concentrations of 0, 0.3, 15, 30 and 60 mg Fe L^−1^ and 0, 0.1, 1, 10, 30 and 60 mg Mn L^−1^ in a final volume of 150 mL/flask. All the procedures were performed in a laminar-flow hood. The stock solutions of the different metal salts were prepared in ultrapure water and filtered through 0.22 µm nylon syringe filters (Filtrilo, São Paulo, Brazil) before use. The pH of media was maintained at 7.4 (using 1 M citric acid or K_2_HPO_4_) when necessary. Flasks were kept for four days in the growth chamber under the same conditions mentioned above. To exclude the possible effect of added SO_4_ and Cl from the iron and manganese salts, a parallel experiment was performed, and the species were treated with a calculated amount of filtered (0.22 µm) K_2_SO_4_ or KCl solution corresponding to ~103.20 mg SO_4_ L^−1^ and ~77.45 mg Cl L^−1^, which corresponded to the highest amount of SO_4_ and Cl added to the media under Fe- and Mn treatments, respectively. Metal concentrations were chosen based on their highest concentrations in water at the time of the species sampling (up to 0.38 mg Fe L^−1^ and up to 0.15 mg Mn L^−1^) and the maximum permissive value by Brazilian legislation (0.3 mg Fe L^−1^ and 0.1 mg Mn L^−1^) [43]. Concentrations higher than those values were tested to observe the metal tolerance of the species and may also simulate metal concentrations in water in the river just after the dam rupture. A total of six replicates per treatment were performed.

### 4.3. NaCl and Metal (Fe and Mn) Combined Effects 

The combined effect of salinity and Fe or Mn was investigated in cells exposed to 0, 0.025, and 5.493 g NaCl L^−1^. These concentrations were chosen based on average chloride concentrations observed in the water of the river at the date of periphyton sampling (15 mg Cl L^−1^) and in the highest Na concentration found along the Doce River (3,330 mg Na L^−1^) (Linhares, Espírito Santo, Brazil) [28], which represents a moderately saline water for freshwater [44]. NaCl concentrations were combined with the highest Fe or Mn concentrations observed in the site of sampling (0.38 and 0.15 mg L^−1^, respectively) and to the metal concentration leading to a 10% decrease on cell density (EC_10_). EC_10_ concentrations were calculated based on the results obtained in the previous described experiment. The EC_10_ was chosen since the concentrations for the observed EC_50_ are not environmentally realistic in river waters even in those from mining areas. The pH of solutions was checked and adjusted at 7.4 (using 1 M citric acid or K_2_HPO_4_) when necessary. Cultures (*n* = 6) were kept for four days in the growth chamber under the same conditions mentioned above.

### 4.4. Evaluation

Initial concentrations (Ci) of Mn, Fe, and NaCl (evaluated by Na concentration) were checked by collecting samples (1 mL) of the growth media at the beginning of the experimental time. After centrifugation (16,200× *g*, 5 min) to eliminate cells [45], supernatant was collected, filtered, and the metals were quantified using inductively coupled plasma-mass spectrometry (ICP-MS Varian 720-ES). To guarantee the quality control procedures, standard solutions were prepared by the dilution of certified standard material (BCR 414 Plankton, Community Bureau of Reference, Geel, Belgium), and these solutions were used to compare to measured values.

Optical density was used to estimate biomass concentration in the cultures (*n* = 6). For this, we previously used a concentrated culture for which we determined the cell density by direct counting using an optical microscope and a Sedgewick-Rafter counting chamber. Then, we performed a serial dilution resulting in eight different densities to obtain a suitable standard curve in which the lower values were smaller than those we started the experiments with. Data of direct counting were compared to those of the absorbance obtained for OD at 750 nm [46] using a spectrophotometer (Spectronics 20-Genesys, Thermo Scientific, Houston, TX, USA). 

Chlorophyll *a* fluorescence was investigated by using a pulse-amplitude modulated (PAM) fluorometer (model PAM−2500, Walz, Effeltrich, Germany). For that purpose, cultures were pre-acclimated to dark for 15 min. Then, the cultures (*n* = 6) were mixed, and 25 mL sub-samples were collected and filtered in the dark using microfiber filters (grade GF/F−0.45 µm, Whatman Ltd.) and inserted immediately in the leaf clip of the PAM fluorometer. The maximal PSII photochemical yield (Φ_M_) was calculated following Maxwell et al. [47]. For the cyanobacteria, in order to obtain the real F_M_ value, 3-(3,4-dichlorophenyl)−1,1-dimethylurea (DCMU) was added after the determination of the Fo level [48].

For biochemical evaluations, the samples from each Erlenmeyer flask (*n* = 3) were divided into 50 mL Falcon tubes and filtered using microfiber filters (grade GF/F−0.45 µm, Whatman Ltd.) in a total of two filters/flask. Filters were frozen in liquid nitrogen and kept at −20 °C until analyses. Half of the filter (*n* = 3) from each flask was used for antioxidant enzyme evaluation. Extracts were obtained from fresh material collected on filters with a 50 mM potassium phosphate buffer (pH 7.8), containing 100 mM EDTA, 1 mM *L*-ascorbic acid, and 2% PVP (*m*/*v*). The protein content was determined by the Bradford method [49]. Superoxide dismutase (SOD; EC 1.15.1.1) was evaluated following [50]; catalase (CAT; EC 1.11.1.6) was evaluated following [51]; glutathione reductase (GR; EC 1.6.4.2) was determined according to [52] and glutathione-S-transferase (GST; EC 2.5.1.18) assay was performed according to [53]. The other part of filters (*n* = 3) was used to evaluate malondialdehyde concentration (MDA; lipid peroxidation) following [54].

To investigate metal removal, 125 mL samples were collected, centrifuged (16,200× *g*, 5 min) and the supernatants and pellets were separately stored at −20 °C until analyses [45]. The final metal concentration in the culture media was measured in the supernatant (*C_f_*) and the removed metal was calculated as the difference between its initial and final concentrations in the medium.

$$Total\;removed\;metal=C_{i}-C_{f}$$

For measuring the extracellularly bound metal content (*C*_extra_), the pellets were lyophilized, washed in 5 mL of 2 mmol EDTA for 10 min to remove the cell surface bound or precipitated metals and the metals were evaluated in the supernatant after centrifugation (16,200× *g*, 5 min). The amount of intracellularly accumulated metals was calculated by deduction of the extracellularly bound amounts from the calculated total amounts [45]. Metal evaluation was proceeded using ICP-MS (Varian 720-ES). The accuracy of the elemental analyzes was confirmed by carrying standard reference materials through analytical processes. Each batch analyzed included reference material (BCR 414 Plankton, Community Bureau of Reference) with known levels of metals, as well as one blank sample for quality control. The total Fe and Mn recovery in the reference material was 87.5% and 81.2%, respectively. Metal concentrations observed in cell filtered samples were extracted from those observed in samples without cells. The metal removal efficiency was calculated as follows:

$$Removal\;efficiency=100-\left(\frac{C_{f}}{C_{i}}\times 100\right)$$

where *C_f_* and *C_i_* is the final and initial concentration of the metal observed in mg L^−1^ at the growth media.

### 4.5. Statistical Analyses

The effective concentrations causing 10% (EC10) or 50% (EC50) photosynthetic reductions were determined from the nonlinear least square fits, using an inverse regression curve according to van der Heever and Grobbelaar [55].

Statistical analyses were performed using JMP 13.0 software (SAS Institute Inc., Cary, NC, USA). The results were expressed as the average of three replicates. Data were tested for normality (Shapiro–Wilk) and homoscedasticity (Bartlett) and then statistically evaluated. Data from the assays with Fe and Mn were evaluated using one-way analysis of variance (ANOVA) and the means were compared using the post hoc Tukey test at 5% level of probability. Data from assays in which the cyanobacteria were submitted to the factorial treatments with NaCl and Fe/Mn were evaluated using two-way ANOVA. Interactions between NaCl and Fe/Mn were included in the model. When differences were detected by ANOVA, the means were compared by the post hoc Tukey test (significance at *p* < 0.05).

## 5. Conclusions

*S. elongatus* and *C. infusionum* are periphytic algal and cyanobacterial species showing great tolerance to metal (Fe and Mn) as well as salinity (NaCl) stress. These species employed different strategies to accumulate Fe and Mn which were reflected in their metal tolerances: while *C. infusionum* restrained the transport of metals to internal compartments of cells (avoiding their deleterious effects on metabolism), *S. elongatus* were less selective allowing greater entrance of Fe and Mn, which resulted in oxidative damage and related decreases on photosynthesis and growth. However, deleterious effects of the metals were only observed in extremely high concentrations of Fe and Mn, which are not representative of the realistic concentrations occurring in water of rivers from where these species were sampled. However, both species showed a capacity for the removal of Fe and Mn, with the exception of *S. elongatus* when Mn concentrations were greater than 30 mg L^−1^ (a situation rarely observed). Increased water salinity did not affect the growth, metabolism, or metal-removal efficiency of the studied microorganisms. Due to their salt and metal tolerance, *S. elongatus* and *C. infusionum* could be used in new microorganism-based wastewater bioremediation technologies. The treatment of wastewater with high salt content, for instance, presents a complicate task for wastewater treatment professionals since it cannot be introduced either into surface waters or into general wastewater treatment systems without pre-treatment. In this context, *S. elongatus* and *C. infusionum* can be used for metal removal but the NaCl removal ability of these species must be evaluated.

## Figures and Tables

**Table 1:** Initial concentration in the media (C_i_, mg L^−1^), total removal (TR, mg L^−1^), extracellular concentration (C_extra_, ng 10^−6^ cells), intracellular concentration (C_intra_, ng 10^−6^ cells) and metal-removal efficiency (RE, %) in *Synechococcus elongatus and Chlorococcum infusionum cells exposed to increased concentrations of Fe (mg L^−1^)* for 96 h. Data represent means of three replicates. Means ± SD of three replicates. Means followed by the same letter, in the line and within each metal and species, did not differ significantly (*p* > 0.05) by the post hoc Tukey test. nd—not detected.

.	Fe (mg L^−1^)					
	0	0.3	3	15	30	60
C_i_	8.2 × 10^−4^ ± 0.4 × 10^−4 f^	0.31 ± 0.02 ^e^	3.12 ± 0.32 ^d^	15.01 ± 0.10 ^c^	31.25 ± 1.16 ^b^	60.01 ± 1.75 ^a^
	***Synechococcus** **elongatus***					
TR	0.00 ± 0.00 ^f^	0.13 ± 0.00 ^e^	1.09 ± 0.17 ^d^	4.92 ± 0.67^c^	16.87 ± 0.83^b^	21.85 ± 1.72 ^a^
C_extra_	0.03 ± 0.00 ^d^	15.86 ± 4.65 ^cd^	90.36 ± 18.17 ^bc^	474.90 ± 250.64 ^b^	5974.99 ± 2925.35 ^a^	9811.92 ± 3940.66 ^a^
C_intra_	0.08 ± 0.00 ^d^	29.64 ± 6.55 ^cd^	159.14 ± 49.99 ^bc^	967.28 ± 155.97 ^b^	9196.76 ± 3632.58 ^a^	16592.16 ± 6097.36 ^a^
RE	46.1 ± 4.5 ^ab^	42.1 ± 2.7 ^bc^	35.1 ± 5.6 ^c^	32.8 ± 4.4 ^d^	56.1 ± 2.7 ^a^	36.4 ± 2.8 ^bc^
	***Chlorococcum infusionum***					
TR	0.00 ± 0.00 ^f^	0.16 ± 0.00 ^e^	1.39 ± 0.11 ^d^	8.65 ± 1.20 ^c^	21.97 ± 0.87 ^b^	33.33 ± 2.50 ^a^
C_extra_	0.05 ± 0.00 ^f^	14.37 ± 2.21 ^e^	109.58 ± 6.69 ^d^	1124.67 ± 270.27 ^c^	3629.25 ± 240.24 ^b^	10673.54 ± 1068.90 ^a^
C_intra_	0.11 ± 0.02 ^f^	27.14 ± 4.30 ^e^	189.39 ± 9.01 ^d^	905.70 ± 100.47 ^c^	2201.31 ± 278.58 ^b^	5989.91 ± 641.00 ^a^
RE	66.3 ± 8.6 ^ab^	53.7 ± 1.2 ^bc^	44.7 ± 3.6 ^c^	57.6 ± 8.0 ^bc^	73.2 ± 2.9 ^a^	55.5 ± 4.1 ^bc^

**Table 2:** Initial concentration in the media (C_i_, mg L^−1^), total removal (TR, mg L^−1^), extracellular concentration (C_extra_, ng 10^−6^ cells), intracellular concentration (C_intra_, ng 10^−6^ cells), and metal-removal efficiency (RE, %) in *Synechococcus elongatus and Chlorococcum infusionum cells exposed to increased concentrations of Mn (mg L^−1^)* for 96 h. Data represent means of three replicates. Means ± SD of three replicates. Means followed by the same letter, in the line and within each metal and species, did not differ significantly (*p* > 0.05) by the post hoc Tukey test. nd—not detected.

	Mn (mg L^−1^)					
	0	0.1	1	10	30	60
**C_i_**	n.d	0.11 ± 0.00 ^e^	1.02 ± 0.23 ^d^	10.06 ± 0.39 ^c^	30.06 ± 0.33 ^b^	60.02 ± 0.13 ^a^
	***Synechococcus** **elongatus***					
**TR**	-	0.08 ± 0.00 ^c^	0.76 ± 0.18 ^bc^	4.01 ± 0.25 ^a^	2.42 ± 1.56 ^ab^	2.15 ± 0.57 ^ab^
**C_extra_**	-	6.97 ± 0.69 ^c^	102.60 ± 13.90 ^bc^	1394.39 ± 140.95 ^b^	8557.21 ± 5786.71 ^a^	5005.08 ± 1672.92 ^a^
**C_intra_**	-	14.82 ± 1.46 ^c^	179.57 ± 40.97 ^bc^	3712.49 ± 875.41 ^ab^	1293.15 ± 8082.12 ^a^	14,067.02 ± 3480.82 ^a^
**RE**	-	79.2 ± 5.8 ^a^	65.7 ± 7.9 ^b^	46.7 ± 2.6 ^c^	2.3 ± 0.2 ^d^	1.1 ± 0.1 ^e^
	***Chlorococcum infusionum***					
**TR**	-	0.08 ± 0.00 ^e^	0.90 ± 0.05 ^d^	5.38 ± 0.22 ^c^	14.11 ± 1.64 ^b^	24.90 ± 1.20 ^a^
**C_extra_**	-	6.84 ± 1.26 ^e^	73.69 ± 9.72 ^d^	751.05 ± 65.43 ^c^	3485.03 ± 789.47 ^b^	8831.65 ± 435.99 ^a^
**C_intra_**	-	10.06 ± 1.19 ^e^	119.12 ± 21.25 ^d^	557.52 ± 13.13 ^c^	1717.64 ± 201.72 ^b^	4778.58 ± 692.02 ^a^
**RE**	-	72.5 ± 4.6 ^bc^	88.4 ± 4.3 ^a^	53.5 ± 2.4 ^bc^	46.9 ± 5.4 ^cd^	41.4 ± 2.0 ^d^

**Table 3:** F values and two-way ANOVA results for the effects of Fe/Mn and NaCl at different concentrations on cell density, maximal PSII photochemical yield (Φ_M_), and metal-removal efficiency (RE) on Synechococcus elongatus *and* Chlorococcum infusionum *cells. Values represent the means of three replicates*.

Anova F Values	D.F	Cell Density		Φ_M_		RE	
		*S. elongatus*	*C. infusionum*	*S. elongatus*	*C. infusionum*	*S. elongatus*	*C. infusionum*
Fe	2	8.11 **	19.46 ***	13.14 ***	0.69	28.53 ***	13.20 ***
NaCl	2	1.90	0.60	1.07	0.33	0.06	2.13
Fe × NaCl	4	0.87	1.15	4.11 *	1.40	3.85 *	4.60 *
Comparison of means ^$^							
Fe (mg L^−1^)							
0		2.91 ^a^	3.39 ^b^	0.54 ^a^	0.61	43.57 ^a^	66.75 ^a^
0.38		2.95 ^a^	4.03 ^a^	0.52 ^b^	0.61	44.95 ^a^	68.37 ^a^
EC_10_^$$^		2.47 ^b^	3.97 ^a^	0.42 ^b^	0.60	28.03 ^b^	53.14 ^b^
NaCl (g L^−1^)							
0		3.03	3.86	0.53	0.61	40.85	58.26
0.025		2.72	3.80	0.52	0.61	35.27	65.04
5.493		2.58	3.73	0.44	0.60	40.41	64.97
Mn		32.33 ***	110.58 ***	19.51 ***	17.95 ***	0.20	59.64 ***
NaCl		7.58 **	2.52	0.07	3.56	0.70	2.48
Mn × NaCl		0.27	2.63	0.15	1.29	0.81	1.33
Comparison of means ^$^							
Mn (mg L^−1^)							
0		2.91 ^b^	17.88 ^a^	0.54 ^a^	0.61 ^a^	-	-
0.3		3.62 ^a^	18.22 ^a^	0.54 ^a^	0.62 ^a^	61.72	78.50 ^a^
EC_1 0_^$$^		2.47 ^c^	5.88 ^b^	0.27 ^b^	0.58 ^b^	58.05	36.10 ^b^
NaCl (g L^−1^)							
0		3.30 ^a^	16.33	0.46	0.61	42.99	60.07
0.025		2.94 ^ab^	10.44	0.45	0.60	41.24	57.41
5.493		2.75 ^b^	15.22	0.45	0.60	35.53	54.41

D.F.: degrees of freedom; * significant *p* < 0.05; ** significant *p* < 0.01; *** significant *p* < 0.001. ^$^ Treatment means from ANOVA. Values followed by the same letter, within the same source of variation, are not significantly different (*p* < 0.05) by Tukey (for all variables from Fe × NaCl treatment, and cell density and Φ_M_ from Mn × NaCl treatment) or Student *t*-test (Mn-RE). ^$$^ EC10 = 15.2 and 53.9 mg Fe L^−1^ and 31.6 and 2.5 and 53.2 mg Mn L^−1^ for S. elongatus and C. infusionum, respectively.

## Data Availability

The data that support the findings of this study are available from the corresponding author upon reasonable request.
